# Clinical course of chronic hepatitis B patients who were off-treated after lamivudine treatment: analysis of 138 consecutive patients

**DOI:** 10.1186/1743-422X-9-239

**Published:** 2012-10-18

**Authors:** Young-Joo Jin, Kang Mo Kim, Dong-jun Yoo, Ju Hyun Shim, Han Chu Lee, Young-Hwa Chung, Yung Sang Lee, Dong Jin Suh

**Affiliations:** 1 Department of Internal Medicine, Asan Liver Center, Asan Medical Center, University of Ulsan College of Medicine, 88, Olympic-ro 43-gil, Songpa-gu, Seoul, 138-736, South Korea

**Keywords:** Chronic hepatitis B, Lamivudine cessation, Virologic relapse, Biochemical breakthrough, Hepatitis flare

## Abstract

**Background/aims:**

Little is known about the long-term outcome of chronic hepatitis B (CHB) patients who discontinued antiviral therapy. We intended to analyze the long-term outcome of CHB patients who discontinued lamivudine therapy and to evaluate predictors for post-treatment outcome.

**Material/methods:**

From 2007 to 2008, 138 lamivudine off-treated CHB patients with alanine aminotransferase normalization were consecutively enrolled. Post-treatment virologic relapse, biochemical breakthrough, hepatitis flare, and retreatment results were retrospectively analyzed.

**Results:**

Among 138 patients, 102 were initially HBeAg-positive at the start of lamivudine treatment. Virologic relapse, biochemical breakthrough, and hepatitis flare were observed in 45.2, 52.9, and 12.7% of HBeAg-positive and 29.4, 30.6, and 8.3% of HBeAg-negative patients during the median follow-up of 28 and 30 months, respectively. The cumulative virologic relapse and biochemical breakthrough rates were significantly lower in patients with HBV DNA <50 copies/mL than 50-10^4^ copies/mL at lamivudine cessation. Hepatitis flare was observed in 4.8 and 11.8% of HBeAg-positive and HBeAg-negative patients with HBV DNA <50copies/mL, respectively. Thirty-eight among 138 patients received retreatment and most of them achieved biochemical (37/38) and virologic response (35/38) within 1 year of retreatment. Undetectable serum HBV DNA (<50 copies/mL) and young age at lamivudine cessation were inversely associated with virologic relapse. Undetectable HBV DNA at cessation, female, and initial HBeAg-negative were inversely associated with biochemical breakthrough.

**Conclusions:**

Post-treatment virologic relapse and biochemical breakthrough incidence were low in patients who achieved undetectable viral titer at lamivudine cessation. Retreatment after biochemical breakthrough or virologic relapse was safe and effective. Intermittent antiviral therapy might be cautiously considered in appropriately selected CHB patients.

## Background

Chronic hepatitis B (CHB) infection is estimated to affect about 350 million persons worldwide [1]. Given that a significant proportion of CHB patients develop the complications of cirrhosis, end-stage liver disease or even hepatocellular carcinoma over time [2,3], lamivudine, which was the firstly approved oral nucleotide analogue (NA) for CHB treatment, has been shown to reduce the risk of liver-related complications with sufficient suppression of viral loads in CHB patients [2,4,5]. However, continued lamivudine therapy cannot always maintain good antiviral responses over time due to inevitable appearance of drug resistance mutation [3,6-8]. Moreover, economic burden of long term lamivudine therapy on an individual patient with HBV is big constraint [9,10], and further pregnancy may zeopardize the long term lamivudine therapy and its outcome [11].

Trials for discontinuation of sustained lamivudine therapy have been issued [12,13]. Currently, several CHB treatment guidelines recommend that lamivudine could be discontinued after consolidation therapy of at least 6-12 months following achievement of undetectable serum HBV DNA level and/or HBeAg loss/seroconversion [3,8,14,15]. However, stopping lamivudine therapy is not always easy since virologic relapse or biochemical breakthrough including hepatitis flare are important concerns both for CHB patients and clinicians [16]. In fact, post-treatment virologic relapse is not rare, but the definite endpoint of lamivudine therapy still remains to be elucidated [12,13,17]. Moreover, little is known about the long-term clinical course of CHB patients who discontinued lamivudine therapy in terms of hepatitis flare and retreatment result, and factors affecting long-term outcomes of patients who discontinued lamivudine therapy are still unclear.

In this retrospective study, we analyzed long-term comprehensive clinical course of consecutive CHB patients who were off-treated after sufficient lamivudine treatment and tried to find out predisposing factors which could influence the clinical outcome of these patients. In addition, we intended to analyze the outcome in re-treated patients after cessation of lamivudine.

## Patients and methods

### Study subjects

**Figure 1:**
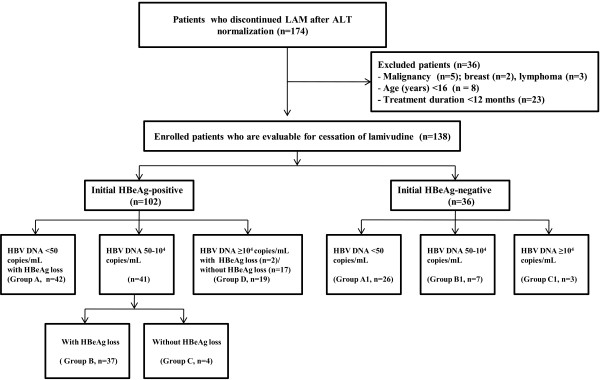
**Flow sheet of enrolloment of the consecutive patients who were off-treated after lamivudine treatment.** LAM, lamivudine; ALT, alanine aminotransferase.

Between January 2007 and December 2008, a total of 174 CHB patients discontinued lamivudine after at least 12 months treatment at our institution and they showed normal alanine aminotransferase (ALT) level at the cessation of lamivudine. None had previously been received other nucleot(s)ide analogues or interferon-alpha before lamivudine treatment. We tried to avoid selection bias by enrolling all the consecutive patients who discontinued lamivudine therapy regardless of the achievement of virologic response in a given period (Figure 1). Thirty-six out of a total of 174 patients were excluded since they had a history of malignancy [breast (n = 2) and lymphoma (n = 3)], they were younger than 16 years (n = 8), or they administrated lamivudine for less than 12 months due to poor compliance (n = 23). The remaining 138 consecutive patients were finally enrolled in this retrospective analysis. ALT normalization was defined by observation of ALT level less than 40 IU/L at the time of lamivudine cessation because this study cohort was retrospectively collected.

This study protocol was approved by the Institutional Review Board at Asan Medical Center, Seoul, Korea (protocol number: 2010-0425).

### Classification of patients with initially HBeAg-positive and HBeAg-negative

Patients were classified based on serum HBV DNA concentration and/or HBeAg status at cessation of lamivudine therapy (Figure 1). In initially HBeAg-positive patients, patients who showed serum HBV DNA <50 copies/mL with HBeAg loss were classified as Group A, patients who showed serum HBV DNA 50-10^4^ copies/mL with HBeAg loss as Group B, and patients who could not achieve HBeAg loss despite serum HBV DNA 50-10^4^ copies/mL as Group C, and patients who showed sustained serum HBV DNA ≥10^4^ copies/mL irrespective with achievement of HBeAg loss as Group D.

In initially HBeAg-negative patients, patients who showed serum HBV DNA <50 copies/mL were classified as Group A1, patients who showed HBV DNA 50-10^4^ copies/mL as Group B1, and patients who showed sustained HBV DNA ≥10^4^ copies/mL as Group C1 (Figure 1).

### Follow-up strategies for post-treatment outcomes

Patients were regularly followed up at a 1-2 month interval for the first 6 months after cessation of lamivudine and thereafter, they were followed up at a 3-6 month interval [15]. Serum HBV DNA level, HBeAg/anti-HBe, and serum ALT level were assayed at every visit after cessation of lamivudine. Measurement of serum HBV DNA level was performed by quantitative real-time PCR using Abbott Real-time HBV quantification kits (detection limit: 50 copies/mL, Abbott Laboratories, USA). For patients whose serum HBV DNA showed sustained elevation above 10^4^ copies/mL despite ALT normalization with or without HBeAg seroconversion, lamivudine-resistant mutations were genotyped using a restriction fragment mass polymorphism method as previously described [18].

### Definition of antiviral response and post-treatment outcomes

Virogic response was defined as decrease in serum HBV DNA level below 10^4^ copies/mL after lamivudine treatment. Serologic response was defined as HBeAg loss/seroconversion after lamivudine treatment in initially HBeAg-positive patients.

Post-treatment outcomes after cessation of lamivudine were categorized as virologic relapse, biochemical breakthrough, hepatitis flare, and HBeAg reversion. Post-treatment virologic relapse was defined as increase in serum HBV DNA level above 10^4^ copies/mL on 2 consecutive tests at least 1 month apart during follow-up period after lamivudine cessation in patients who showed virologic response at the time of lamivudine cessation. Biochemical breakthrough was defined as increase of ALT level above upper limit of normal (ULN, 40 IU/L) on 2 consecutive tests at least 1 month apart during follow-up period after lamivudine cessation in patients who had normalization of ALT at the time of lamivudine cessation. Hepatitis flare was defined as elevation of ALT level more than 10 times the ULN after lamivudine cessation. HBeAg reversion was defined as reappearance of HBeAg during follow-up period after lamivudine cessation in patients who achieved HBeAg loss in initially HBeAg-positive patients. These post-treatment outcomes were analyzed according to classification of patients with initially HBeAg-positive and HBeAg-negative.

### Statistical analyses

Differences between categorical or continuous variables were analyzed using the *chi*-square test, Fisher’s exact test, or Student *t* test. Cumulative post-treatment outcomes were analyzed using Kaplan–Meier method and log-rank test. Multivariate analyses of the potential predictors for virologic relapse and biochemical breakthrough were performed using Cox’s proportional hazard model. A two tailed *P*-value of <0.05 was considered statistically significant.

## Results

### Patient characteristics

The baseline characteristics of all the 138 patients at the time of initiation and cessation of lamivudine therapy are shown in Table 1. There were no significant differences in sex and serum level of ALT, albumin, and total Bilirubin except age between initially HBeAg-positive and HBeAg-negative patients. Median duration of lamivudine treatment was 35.5 months (range, 13-98 months). Before initiation of antiviral treatment, liver cirrhosis was observed in 17 of 138 patients, and none of them had decompensated liver cirrhosis (Table 1).

**Table 1:** Baseline characteristics of all 138 patients at the initiation and cessation of lamivudine treatment

**Characteristics at the initiation of LAM**	**All patients(*n*** **= 138)**	**Initial HBeAg- positive(*n*** **= 102)**	**Initial HBeAg- negative(*n*** **= 36)**	*P*-value*
Age (years)	39 (16-79)	37 (16-67)	45 (25-79)	0.001
Sex (male), *n* (%)	82 (59.4)	59 (57.8)	23 (63.9)	0.560
ALT (IU/L)	204 (41-1476)	214 (41-994)	164 (42-1476)	0.864
Albumin (g/dL)	3.9 (1.8-4.6)	3.9 (1.8-4.6)	4.0 (2.3-4.4)	0.664
Total bilirubin (mg/dL)	1.2 (0.4-22.1)	1.2 (0.4-22.0)	1.2 (0.5-22.1)	0.430
HBV DNA (Log_10_ copies/mL)	7.4 (5.0-10.0)	7.5 (5.0-10.0)	7.4 (5.4-9.1)	0.877
Liver cirrhosis	17 (12.3)	9 (8.8)	8 (22.2)	0.040
**Characteristics at cessation of LAM**	***n =* 138**	***n =* 102**	***n =* 36**	***P*-value**
Age (years)	43 (19-82)	41 (19-69)	48 (28-82)	0.001
ALT (IU/L)	18 (5-38)	19 (7-38)	18 (5-38)	0.696
Albumin (g/dL)	4.1 (2.8-4.7)	4.1 (2.8-4.7)	4.1 (3.4-4.4)	0.518
Total bilirubin (mg/dL)	1.1 (0.5-1.8)	1.1 (0.6-1.8)	1.1 (0.5-1.2.0)	0.297
HBV DNA (copies/mL), n (%)†				0.006
<50	68 (49.3)	42 (41.2)	26 (72.2)	
50-10^4^	48 (34.8)	41 (40.2)	7 (19.4)	
10^4^ ≤	22 (15.9)	19 (18.6)	3 (8.3)	
HBeAg loss/seroconversion, *n* (%)	*NA*	82 (80.4)	*NA*	
Treatment duration (month)	35.5 (13-98)	35.6 (13-98)	35.1 (14-80)	0.333

Data were presented as median (range).

ALT, alanine aminotransferase; LAM, lamivudine; *NA*, not applicable.

^†^Serum HBV DNA at cessation of lamivudine.

* P-value for difference between the Initial HBeAg-positive and HBeAg-negative groups.

In 102 initially HBeAg-positive patients, 83 (81.4%) patients achieved serum HBV DNA <10^4^ copies/mL at cessation of lamivudine (Figure 1). Of these 83 patients, 42 (50.6%) patients showed serum HBV DNA <50 copies/mL with HBeAg loss (Group A), 37 (44.6%) showed serum HBV DNA 50-10^4^ copies/mL with HBeAg loss (Group B), and 4 (4.8%) could not achieve HBeAg loss despite serum HBV DNA 50-10^4^ copies/mL (Group C). Nineteen (18.6 ) out of 102 HBeAg-positive patients showed sustained serum HBV DNA ≥10^4^ copies/mL with achievement of HBeAg loss (n = 2) and without it (n = 17) (Group D) and the remaining 83(81.4%) received the extended therapy for 12 months after achievement of serum HBV DNA <50 (n = 42, 41.2%) and 50-10^4^ copies/mL (n = 41, 40.2%).

Thirty-seven patients of group B discontinued lamivudine despite their HBV DNA level (50-10^4^ copies/mL) due to the economic burden for continuous medication, the need for pregnancy (n = 3), or lamivudine-resistance mutation: M204V (n = 3) and M204I (n = 2) with L180M; M204I without L180M (n = 1). Twenty-two patients of group C and D discontinued lamivudine after achievement of ALT normalization for more than 1 year due to the economic burden for continuous medication or lamivudine-resistance mutation: M204V (n = 11), M204I (n = 1), and M204V/M204I (n = 1) with L180M; M204V without L180M (n = 1).

In 36 initially HBeAg-negative patients, 26 (72.2%) patients showed serum HBV DNA <50 copies/mL (Group A1), 7 (19.4%) patients showed HBV DNA 50-10^4^ copies/mL (Group B1), and the remaining 3 (8.3%) patients showed sustained HBV DNA ≥10^4^ copies/mL (Group C1) at cessation of lamivudine (Figure 1). Thirty-three (91.7%) out of 36 HBeAg-negative patients received extended therapy for 12 months after achievement of serum HBV DNA <50 (n = 26) and 50-10^4^ copies/mL (n = 7).

Seven patients of group B1 discontinued lamivudine after achievement of low viral loads although their serum HBV DNA level was not less than 50 copies/mL due to the economic burden for continuous medication or lamivudine-resistance mutation (M204V with L180M, n = 3). Three patients of group C1 discontinued lamivudine after achievement of ALT normalization for more than 1 year due to the economic burden for sustained medication or lamivudine-resistance mutation (M204V with L180M, n = 1).

### Clinical course of initially HBeAg-positive patients after lamivudine cessation

**Figure 2:**
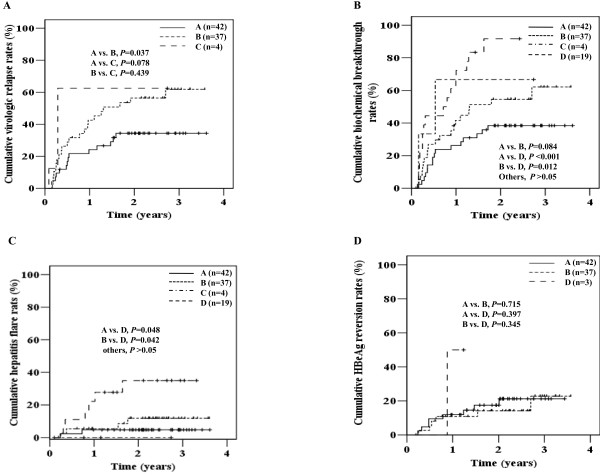
**Clinical courses of initial HBeAg-positive patients after lamivudine cessation.** Virologic relapse (**A**), biochemical breakthrough (**B**), hepatitis flare (**C**), and HBeAg reversion (**D**). Patients were grouped according to serum HBV DNA level and HBeAg status at cessation of lamivudine treatment: Group A, HBV DNA <50 copies/mL with HBeAg loss; Group B, HBV DNA 50-10^4^ copies/mL with HBeAg loss; Group C, HBV DNA 50-10^4^ copies/mL without HBeAg loss; Group D, HBV DNA ≥10^4^ copies/mL irrespective HBeAg loss.

Among 102 initially HBeAg-positive patients, analysis of virologic relapse was available in 83 patients who achieved serum HBV DNA <10^4^ copies/mL at cessation of lamivudine, and biochemical breakthrough and hepatitis flare were analyzed in all 102 patients. During the median follow-up period of 28 months after lamivudine cessation, virologic relapse, biochemical breakthrough, and hepatitis flare were observed in 44.6, 52.9, and 12.7% of patients, respectively. The 3-year cumulative virologic relapse was significantly less common in patients with serum HBV DNA <50 copies/mL than patients with HBV DNA 50-10^4^copies/mL (group A vs. B: 34.4 vs. 60.9%, *P* = 0.037) (Figure 2A).

The 3-year cumulative biochemical breakthrough rates in patients of group A, B, C, and D were 38.5, 62.6, 66.7, and 90.9%, respectively. The patients with serum HBV DNA >10^4^ copies/mL showed significantly higher biochemical breakthrough rate than the other patients (group A vs. D, *P* <0.001; group B vs. D, *P* = 0.012) (Figure 2B). The 3-year cumulative hepatitis flare rate was significantly higher in patients with serum HBV DNA >10^4^ copies/mL than the other patients (group A vs. D: 4.8 vs.21.8%, *P* = 0.048*;* B vs. D: 11.9 vs.21.8%, *P* = 0.042; C *vs.* D: 0% vs. 21.8%, *P* = 0.444; others, *P* > 0.05) (Figure 2C). Hepatitis flare was not observed in patients of group C although biochemical breakthrough was observed in them.

Analysis of HBeAg reversion was available in 82 patients who achieved HBeAg loss at cessation of lamivudine. There was no significant difference in the 3-year cumulative HBeAg reversion rate between group A and B (21.3 vs.23.04%, *P* = 0.715), and 1 of 3 patient of group D showed HBeAg reversion at 11 months after cessation of lamivudine (Figure 2D).

**Figure 3:**
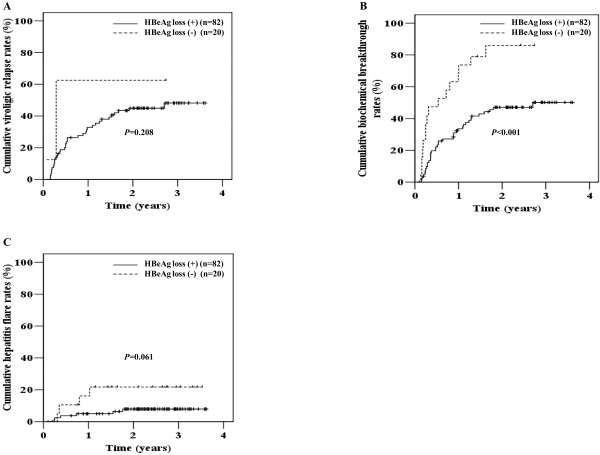
**Clinical courses after lamivudine cessation in initial HBeAg-positive patients according to serologic response.** Virologic relapse (**A**), biochemical breakthrough (**B**), and hepatitis flare (**C**).

Biochemical breakthrough was significantly higher in patients who discontinued lamivudine after achievement of HBeAg loss than who did not (*P* < 0.001) (Figure 3B). However, virologic relapse and hepatitis flare did not show significant association with serologic response at cessation of lamivudine (*P* = 0.208 and *P* = 0.061, respectively) (Figure 3A and 3C).

### Clinical course of initially HBeAg-negative patients after lamivudine cessation

Among 36 initially HBeAg-negative patients, analysis of virologic relapse was available in 33 patients with serum HBV DNA <10^4^ copies/mL at cessation of lamivudine. Biochemical breakthrough and hepatitis flare were analyzed in all 36 patients.

**Figure 4:**
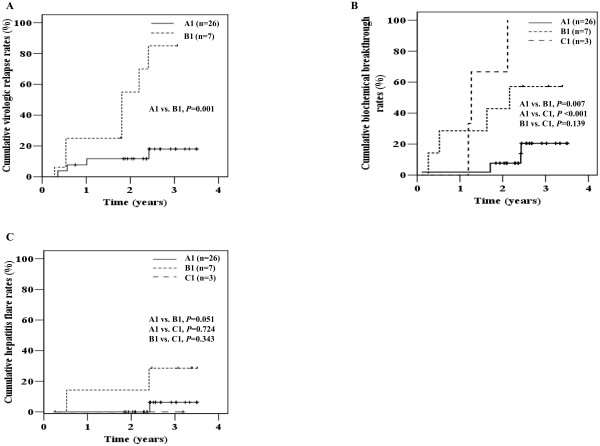
**Clinical courses of initial HBeAg-negative patients after lamivudine cessation.** Virologic relapse (**A**), biochemical breakthrough (**B**), and hepatitis flare (**C**). Patients were grouped according to serum HBV DNA level at cessation of lamivudine treatment: Group A1, HBV DNA <50 copies/mL; Group B1, HBV DNA 50-10^4^ copies/mL; Group C1, HBV DNA ≥10^4^ copies/mL.

During a median follow-up of 30 months after cessation of lamivudine, virologic relapse, biochemical breakthrough, and hepatitis flare were observed in 27.3, 30.6, and 11.8% of patients, respectively. The 3-year cumulative virologic relapse was significantly less common in patients with HBV DNA <50 copies/mL than those with HBV DNA 50-10^4^ copies/mL at the cessation of lamivudine (group A1 vs. B1: 18.0 vs. 82.1%, *P =* 0.001) (Figure 4A). The 3-year cumulative biochemical breakthrough rate was significantly lower in patients with HBV DNA <50 copies/mL than those with HBV DNA 50-10^4^ copies/mL at cessation of lamivudine (group A1 vs. B1: 20.5 vs. 57.1%, *P* = 0.019) (Figure 4B). There was no significant difference in the 3-year cumulative hepatitis flare between groups A1 and B1 (6.3 vs. 33.3%, *P =* 0.051) (Figure 4C). Hepatitis flare was not observed in group C.

### Clinical course of retreated patients

Among 138 patients, 38 patients were retreated with oral NAs due to virologic relapse or ALT elevation (≥2 x ULN) (Table 2) and of these, 12 (31.6%) patients showed hepatitis flare (≥10 x ULN). The median time interval between lamivudine cessation and hepatitis flare was 8 months (range, 3-29 months). Eight (66.7%) of 12 patients with hepatitis flare showed virologic relapse at the time of retreatment and 4 (33.3%) showed sustained serum HBV DNA >10^4^ copies/mL from the time of lamivudine cessation. Fifteen (57.7%) of 26 patients without hepatitis flare showed virologic relapse at the time of retreatment and the remaining 11 (19.2%) patients revealed sustained serum HBV DNA >10^4^ copies/mL from the time of lamivudine discontinuation.

**Table 2:** Characteristics of the all 38 retreated patients with oral antiviral agents

**Characteristics**	**Total (*n*** **= 38)**	**Initial HBeAg-positive (*n*** **= 31)**	**Initial HBeAg- negative (*n*** **= 7)**
*At the time of retreatment*			
ALT (IU/L)	264 (81-895)	233 (83-895)	311 (81-575)
>2 x and ≤10 x ULN, *n* (%)	26 (68.4)	21 (67.7)	5 (71.4)
>10 x ULN, *n* (%)	12 (31.6)	10 (32.3)	2 (28.6)
HBV DNA (Log_10_copies/mL)	8.1 (5.3-9.7)	8.1 (5.3-9.7)	8.0 (5.8-8.9)
Time to hepatitis flare (months) *	8 (3-29)	7 (3-21)	18 (6-29)
Follow-up after cessation (months)	29 (2-43)	28 (2-43)	30 (3-42)
*At the last follow-up after retreatment*			
ALT normalization, *n* (%)	37 (97.4)	31 (100)	6 (85.7)
Time to ALT normalization (months)	4 (1-21)	4 (1-21)	3 (3-26)
Serum HBV DNA level, *n* (%)			
<50 copies/mL	24 (63.2)	19 (61.3)	5 (71.4)
50-10^4^ copies/mL	11 (28.9)	9 (29.0)	2 (28.6)
≥10^4^ copies/mL	3 (7.9)	3 (9.7)	0
Time to serum HBV DNA level (months)			
<50 copies/mL	10 (3-33)	12 (1-33)	9 (3-26)
50-10^4^ copies/mL	8 (1-31)	8 (1-31)	11 (3-26)
Hospitalization, *n* (%)	1 (2.6)	1 (3.2)	0 (0)
Retreatment drugs:			
ETV/LAM/CLV/ADV, n (%)	30/4/3/1	27/2/2/0	3/2/1/1
	(78.9/10.5/7.9/2.6)	(87.1/6.5/6.5/0)	(42.9/28.6/14.3/14.3)
Follow-up after retreatment (mo)	18 (2-39)	18 (2-39)	17 (6-33)

Data were presented as median (range).

*ADV*, adefovir; *ALT*, alanine aminotransferase; *CLV*, clevudine; *ETV*, entecavir; *LAM*, lamivudine; *Mo*, month; *ULN*, upper limit normal (40 IU/L).

* Time interval between lamivudine cessation and hepatitis flare.

Thirty (78.9%) of the retreated 38 patients received entecavir. The other patients received lamivudine (n = 4), clevudine (n = 3), and adefovir (n = 1) (Table 2). Lamivudine-resistant mutation at the time of retreatment in patients who were retreated with entecavir, lamivudine, clevudine, and adefovir was detected in 4, 2, 2, and 1 patients, respectively. In 2 of 4 patients who were retreated with lamivudine, lamivudine resistant mutation was detected after 10 days of antiviral retreatment, and they were finally retreated with combination therapy of lamivudine and adefovir based on the result of lamivudine resistant mutation. During a median follow-up of 18 months after antiviral retreatment, biochemical and virologic responses were observed in 97.4 and 92.1% of retreated patients, respectively within median 1 year of retreatment. All of the 12 patients who experienced hepatitis flare underwent retreatment and all of them achieved ALT normalization and undetectable serum HBV DNA level within median 5 and 9 months, respectively. Only 1 patient required hospitalization at the time of management for hepatitis flare without death.

After cessation of lamivudine therapy, virologic relapse was observed in 48 (34.8%) out of 138 patients. Of these 48 patients, 23 (47.9%) patients were retreated as mentioned earlier and the remaining 25 patients have been closely followed up without retreatment due to sustained ALT normalization (n = 5) and mild serum ALT elevation (<2 x ULN, n = 20). Among 22 patients who showed sustained HBV DNA >10^4^ copies/mL at the cessation of lamivudine, 5 (25%) patients have been closely monitored without retreatment due to sustained ALT normalization (n = 4) and mild serum ALT elevation (<2 x ULN, n = 1).

### Multivariate analysis for predictive factors of virologic relapse and biochemical breakthrough after lamivudine cessation

Of the 116 patients who achieved serum HBV DNA <10^4^ copies/mL at cessation of lamivudine, multivariate analysis for virologic relapse showed that younger age (<40 years) (Hazard ratio [HR] 0.520, *P =* 0.048) and lower serum HBV DNA level (<50 copies/mL) (HR 0.507, *P* = 0.046) at cessation of lamivudine were inversely associated with post-treatment virologic relapse in initially HBeAg-positive patients (Table 3). In initially HBeAg-negative patients, lower serum HBV DNA level (<50 copies/mL) at the cessation of lamivudine was inversely associated with post-treatment virologic relapse (HR 0.186, *P* = 0.015). Younger age (<40 years) at cessation of lamivudine showed no statistical significance (HR 0.276, *P* = 0.236) (Table 3).

**Table 3:** **Multivariate analysis for virologic relapse after lamivudine cessation (*n*** **= 116)**

**HBeAg-positive patients (n = 83)**	**Univariate analysis**			**Multivariate analysis**		
	**HR**	**95% CI**	***P*-value**	**HR**	**95% CI**	*P*-value*
Age at cessation (<40 years)	0.511	0.260-1.005	0.042	0.520	0.264-0.998	0.048
Sex (male)	1.038	0.538-2.003	0.910	-	-	-
Liver cirrhosis, presence	0.923	0.283-3.010	0.894	-	-	-
HBV DNA (Log_10_copies/mL)^+^	0.875	0.657-1.166	0.362	-	-	-
HBeAg loss at LAM cessation	2.503	0.599-10.459	0.209	-	-	-
Treatment duration (months)	0.990	0.974-1.007	0.258	-	-	-
HBV DNA levels (<50 copies/mL)^†^	0.489	0.257-0.971	0.041	0.507	0.261-0.987	0.046
**HBeAg-negative patients (n = 33)**	**HR**	**95% CI**	***P*-value**	**HR**	**95% CI**	***P*-value**
Age at cessation (<40 years)	0.188	0.023-1.510	0.116	0.276	0.033-2.314	0.236
Sex (male)	0.478	0.099-2.307	0.478	-	-	-
Liver cirrhosis, presence	1.689	0.433-6.591	0.451	-	-	-
HBV DNA (Log_10_copies/mL) ^+^	0.509	0.276-1.151	0.354	-	-	-
Treatment duration (months)	0.994	0.950-1.040	0.781	-	-	-
HBV DNA levels (<50 copies/mL)^‡^	0.145	0.038-0.552	0.005	0.186	0.048-0.723	0.015

*C.I*, confidence interval; *HR*, hazard ratio; *LAM*, lamivudine.

^+^Serum HBV DNA level at the initiation of lamivudine treatment.

^‡^Serum HBV DNA level at cessation of lamivudine treatment.

^*^ Cox Proportional Hazards model with a backward elimination method.

Out of a total of 138 patients, initial HBeAg-positive (HR 2.435, *P* = 0.009), and higher serum HBV DNA level at cessation of lamivudine (50-10^4^ copies/mL, HR 1.847, *P* = 0.043; ≥10^4^ copies/mL, HR 5.249, *P* <0.001) were independently associated with post-treatment biochemical breakthrough, but female (HR 0.372, *P* = 0.001) was inversely associated with post-treatment biochemical breakthrough (Table 4). However, there were no significant risk factors for post-treatment hepatitis flare.

**Table 4:** Multivariate analysis for biochemical breakthrough after lamivudine cessation in all 138 patients

**Variable**	**Univariate analysis**			Multivariate analysis*		
	**HR**	**95% C.I**	***P*-value**	**HR**	**95% C.I**	***P*-value**
Age at cessation (≥40 years)	1.001	0.611-1.639	0.998	-	-	-
Sex (female)	0.396	0.225-0.696	0.001	0.372	0.209-0.663	0.001
Initial HBeAg-positive	2.469	1.288-4.735	0.007	2.435	1.249-4.750	0.009
HBV DNA (Log_10_copies/mL) ^+^	1.075	0.866-1.335	0.512	-	-	-
Total treatment duration (months)	1.000	0.987-1.013	0.957	-	-	-
HBV DNA levels (copies/mL) ^‡^						
<50 (control)						
50-10^4^	2.515	1.402-4.514	0.002	1.847	1.018-3.350	0.043
≥10^4^	5.656	2.964-10.793	<0.001	5.249	2.608-9.553	<0.001

C.I, confidence interval; HBV, hepatitis B virus; HR, hazard ratio.

^+^Serum HBV DNA level at initiation of lamivudine treatment.

^‡^Serum HBV DNA level at cessation of lamivudine treatment.

^*^ Cox Proportional Hazards model with a backward elimination method.

## Discussion

We have shown here that the incidence of post-treatment biochemical breakthrough as well as virologic relapse was low in CHB patients who showed undetectable serum HBV DNA (<50 copies/mL) at cessation of lamivudine after sufficient lamivudine treatment. Notably, initially HBeAg-negative or female patients who achieved low viral titer after sufficient lamivudine treatment showed low post-treatment biochemical breakthrough rate. In addition, we found that hepatitis flare was infrequent and antiviral retreatment for CHB patients with hepatitis flare was safe and effective although virologic relapse and biochemical breakthrough were common after cessation of lamivudine therapy.

The present study has distinctive features that we analyzed the comprehensive long-term post-treatment clinical course including hepatitis flare and retreatment results both in initially HBeAg-positive and HBeAg-negative patients. Recently, cessation of antiviral agent discussed in the previous studies [12,13,19,20], but biochemical breakthrough, hepatitis flare, and retreatment outcome were not closely investigated, especially in HBeAg-negative patients. Post-treatment virologic relapse and biochemical breakthrough incidence rates in our study were low in patients who achieved undetectable serum HBV DNA level and were lower than those of previous studies [12,13,21-24]. Moreover, patients in our cohort were regularly and closely followed up after cessation of lamivudine. Considering good results of NA retreatment in relapsed patients, our data could be one evidence for the importance of early detection and early treatment of biochemical breakthrough before hepatitis flare with close follow-up after cessation of lamivudine, especially in patients with predisposing factors for post-treatment biochemical breakthrough as well as virologic relapse.

Despite that achievement of undetectable serum HBV DNA level has been recommended as the endpoint of CHB treatment [3,8,14,15], in fact, many clinicians have used lamivudine continuously due to potential virologic relapse, biochemical breakthrough, or hepatitis flare that can occur after cessation of lamivudine. However, life-long lamivudine treatment can induce drug resistance mutation [6], and Asian patients tended to show less durable serologic response than Western patients despite long-term lamivudine use [22]. Considering these practical clinical settings, our results can provide useful evidence for determining who can stop and when to stop antiviral treatment. Furthermore, our data could strengthen the previous recommendations that lamivudine cessation might be reasonable option both in initially HBeAg-positive and HBeAg-negative patients who achieved low viral titer after sufficient lamivudine treatment.

In terms of hepatitis flare after cessation of antiviral agents, it has been one of the important concerns for clinicians when they stopped antiviral agents in CHB patients. It occurred in about 17-19% during the median follow-up period of 3-6 months after lamivudine cessation in the previous studies [12,16,24-26], but they had limits due to the small cohorts or short-term follow-up period. In contrast, in our study, hepatitis flare was observed only in 8.7% of all enrolled off-treated CHB patients during the median follow-up period of 29 months. Thus, our results might be more acceptable because our outcome was based on the long-term follow-up period. In spite of our results, we have to say that the cessation of antiviral agents should be very cautious in patients with advanced liver disease because hepatitis flare could cause devastating outcome in these patients [16]. There should be sufficient discussion between doctor and patients about risk and benefit of continuation or cessation of antiviral agents, and frequent follow-up scheme should be applied to these patients with advanced liver disease if antiviral agent is cessated.

In multivariate analysis, serum HBV DNA level at the cessation of lamivudine was independent predisposing factor for post-treatment virologic relapse and biochemical breakthrough. In addition, younger age at lamivudine cessation was inversely associated with the post-treatment virologic relapse, which was similar to the previous studies [12,19], suggesting that younger patients have more powerful immune responses than older patients. Interestingly, we found that HBeAg-negative or female patients who achieved low viral titer were inversely associated with post-treatment biochemical breakthrough. The relationship of female with lower risk of post-treatment biochemical breakthrough was a unique finding in our study that has not been discussed in the previous literature. This result might be helpful for female CHB patients of childbearing age. Although we did not find the accurate mechanism for this relationship, hormonal difference might have influence on this association. In addition, given the low incidence rate of post-treatment biochemical breakthrough despite frequent virologic relapse in HBeAg-negative patients of our study, the association of HBeAg-negative patients with low post-treatment biochemical breakthrough might be explained by different virologic virulence or immune response between initially HBeAg-positive and HBeAg-negative patients. However, there need to be further studies to elucidate the mechanism for these relationships in the future.

With regard to antiviral responsiveness in CHB patients who received antiviral retreatment, it is an additional important consideration when clinicians determine the cessation of antiviral therapy. To date, some previous studies have reported about the clinical outcome after retreatment, but the number of patients was smaller without discussion of HBeAg-negative patients, or the follow-up period after retreatment was shorter compared to that of our study [12,19]. Interestingly, we found that antiviral retreatment was safe and effective even in patients with hepatitis flare. Moreover, most re-treated patients achieved virologic and biochemical response rapidly. Therefore, our results suggest that lamivudine might be cautiously discontinued and intermittent antiviral retreatment might be considered in appropriately selected patients.

In terms of HBV genotype, which has been known to be associated with the antiviral response to oral NA(s), several previous researches performed in South Korea have already demonstrated that approximately 100% of the CHB patients in Korea had genotype C [27]. A vast majority of patients in our cohort could be considered to be infected by HBV genotype C. Thus, in the present study, the relationship between HBV genotype and post-treatment outcomes after lamivudine cessation could not be analyzed.

This study had some limitations. First, our study is a retrospective one that selection bias could be possible For example, median serum HBV DNA level was not different between patients with initial HBeAg positive and HBeAg negative patients at the initiation of LAM treatment as shown in Table 1, which was different from the previous reports [28,29]. This finding might be explained by the retrospective design or relatively small number of initial HBeAg negative patients. However, we tried to avoid selection bias by enrolling all the consecutive patients including those who showed serum HBV DNA level >10^4^ copies/mL at cessation of lamivudine in a given period. In addition, prospective trial for off-treatment of NA is difficult to be performed because of potential ethical problem. Second, we could not compare antiviral efficacy among the patients who were retreated by entecavir, lamivudine, clevudine, or adefovir because of the small number of the patients. There need to be further well-designed randomized prospective studies with large sample size to compare the antiviral efficacy among retreatment drugs.

## Conclusions

Post-treatment virologic relapse and biochemical breakthrough incidence rate were low in patients who achieved low viral titer at lamivudine cessation. The incidence of post-treatment biochemical breakthrough was low in initially HBeAg-negative or female patients who achieved low viral load after sufficient lamivudine treatment. Moreover, antiviral retreatment in patients who experienced virologic relapse or hepatitis flare after cessation of lamivudine was safe and effective. Our findings could be clinically useful evidence for cessation of lamivudine therapy despite the retrospective nature of this study. In addition, intermittent antiviral therapy might be cautiously considered in appropriately selected CHB patients.

## Abbreviations

HBV: Hepatitis B virus; CHB: Chronic hepatitis B; HCV: Hepatitis C virus; NA: Nucleos(t)ides analogue; ALT: Alanine aminotransferase; ULN: Upper limit of normal.

## Competing interests

The authors declare that we have no conflict of interest about the paper and financial disclosure in connection with this study does not exist.

## Authors’ contributions

YJ Jin and KM Kim were responsible for the concept and design of the study, the acquisition, analysis and interpretation of the data, and the drafting of the manuscript. DJ Yoo participated in data collecting. HJ Lee helped with the critical revision of the manuscript for important intellectual content. JH Shim, YH Chung, YS Lee, and DJ Suh supervised the study, and provided critical revisions of the manuscript. All authors read and approved the final manuscript.
